# Clinical Medicine and Surgery in Philadelphia

**Published:** 1845-09

**Authors:** 


### THE MEDICAL EXAMINER.

PHILADELPHIA, SEPT., 1845.

### CLINICAL MEDICINE AND SURGERY IN PHILADELPHIA.

From the Journals and Annual Circulars of the Medical Colleges in the principal cities of the United States, we discover an increasing attention to the subject of Clinical Medicine; imitating, in this respect, the course of the great Medical Schools of Europe. In a former number of the Examiner, we mentioned an intention of giving some account of the various institutions of Philadelphia where clinical instruction is afforded, with some notice of their arrangements, and the method of teaching, by which large classes are enabled to partake of their benefits. In our next number we shall endeavour to find room for a fulfilment of this promise. We advert to the subject at the present, merely to correct an error rather extensively promulgated in regard to the clinics held at the Colleges.

Some ten or a dozen years ago, the faculty of Jefferson Medical College established a Dispensary at the College, in order to afford to its students additional opportunities of witnessing diseases, and the effects of remedies. The operation of this has been so satisfactory as not only to induce its continuance and increase, at a very heavy expense, but it has also led to the adoption of a similar plan by the University of Pennsylvania. At these clinics, a great number of patients assemble, afflicted with nearly all the diseases that "flesh is heir to"—those who are able to leave their beds, but are unable to walk, are provided with conveyances, and every thing is done for their relief that skill and humanity can accomplish. The consequence is, as may well be supposed, that the clinics have become favourites, both with the poor and the profession, and hence the crowds who flock to the Colleges on prescribing days—patients to be relieved, and physicians and students to listen to the principles inculcated by the prescribers, and witness the results of their practical application. Now, the mistake has been made, and by some who ought to have known better, of supposing that these clinics are regarded by the institutions at whose expense they are maintained *as substitutes* for attendance on *hospital practice.* Both the University of Pennsylvania, and Jefferson Medical College, require evidence of having attended one season at some approved *hospital,* from every candidate, before he is admitted to examination for a degree. It never has been the intention of either of the Colleges named, to dispense with *hospital* instruction as a pre-requisite to graduation. On the contrary, they desire only to *increase* the opportunities of their pupils for obtaining clinical instruction. And such has actually been the result; for, notwithstanding the popularity of the College clinics, the attendance on the lectures at the *hospitals* in Philadelphia never was so great as within the last three or four years, nor have the exertions of the teachers ever been more unremitting. Whatever *apology* other institutions may think necessary for their course in this matter, it is but just to the two great schools of which we have spoken, that their motives and actions should not be misunderstood in the policy they have adopted and so successfully carried out.

